# Sexual reproduction is the null hypothesis for life cycles of rust fungi

**DOI:** 10.1371/journal.ppat.1010439

**Published:** 2022-05-26

**Authors:** Alistair R. McTaggart, Timothy Y. James, Alexander Idnurm, Robert F. Park, Louise S. Shuey, Michelle N. K. Demers, M. Catherine Aime

**Affiliations:** 1 Centre for Horticultural Science, Queensland Alliance for Agriculture and Food Innovation, The University of Queensland, Ecosciences Precinct, Dutton Park, Queensland, Australia; 2 Department of Ecology and Evolutionary Biology, University of Michigan, Ann Arbor, Michigan, United States of America; 3 School of BioSciences, The University of Melbourne, Melbourne, Victoria, Australia; 4 Plant Breeding Institute, The University of Sydney, Cobbitty, New South Wales, Australia; 5 Queensland Department of Agriculture and Fisheries, Ecosciences Precinct, Dutton Park, Queensland, Australia; 6 Department of Botany and Plant Pathology, Purdue University, West Lafayette, Indiana, United States of America; Boston Children’s Hospital, UNITED STATES

## Abstract

Sexual reproduction, mutation, and reassortment of nuclei increase genotypic diversity in rust fungi. Sexual reproduction is inherent to rust fungi, coupled with their coevolved plant hosts in native pathosystems. Rust fungi are hypothesised to exchange nuclei by somatic hybridisation with an outcome of increased genotypic diversity, independent of sexual reproduction. We provide criteria to demonstrate whether somatic exchange has occurred, including knowledge of parental haplotypes and rejection of fertilisation in normal rust life cycles.

## Background

Rust fungi (Pucciniales and Pucciniomycotina) benefit from asexual and sexual reproduction. Asexual reproduction propagates infectious spore stages, avoids recombination load, and preserves most fit genotypes [1]. Some rust fungi undergo explosive clonal reproduction and have greater economic impact in agriculture than do obligate-outcrossing pathogens in the Ustilaginomycotina, which are mostly nonpathogenic in clonal stages [2].

**Fig 1:**
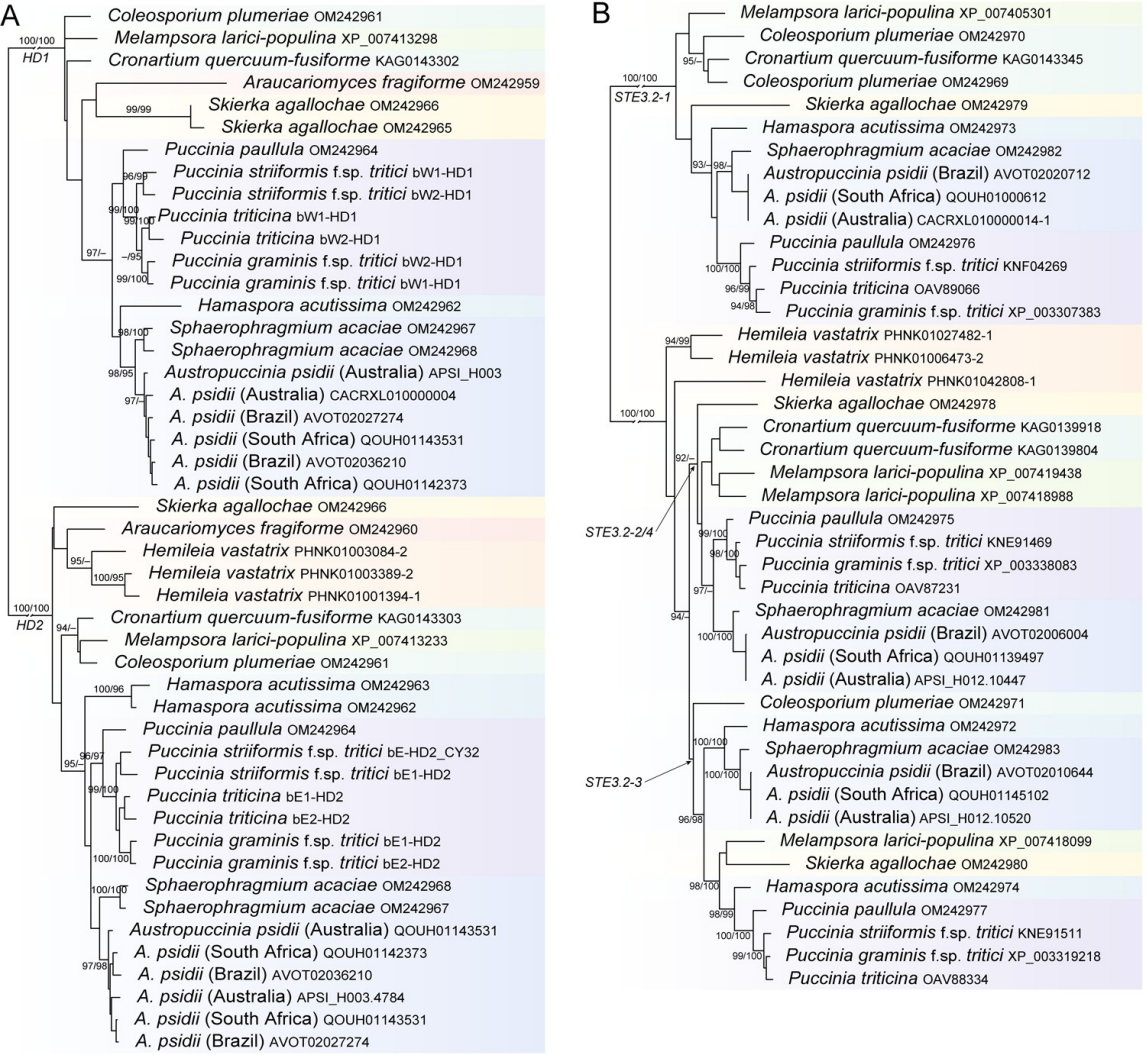
Phylograms of the homeodomain locus **(A)** and pheromone/receptor homologues **(B)** in 8 families of Pucciniales: Araucariomycetaceae, Coleosporiaceae, Melampsoraceae, Phragmidiaceae, Pucciniaceae, Skierkaceae, Sphaerophgramiaceae and Zaghouaniaceae. Genes at the homeodomain locus (*HD1* and *HD2*) are suspected to regulate mating compatibility in rust fungi and are present in all suborders of Pucciniales.

Clonal stages of rust fungi drive boom and bust cycles on annual plants in native ecosystems [3]. Sexual reproduction provides evolutionary innovation to overcome resistance the following season [3], and pathogens and hosts can be at equilibria of virulence/resistance after millennia of coevolution [4]. Clonal stages dominate life cycles of most rust fungi; however, meiosis and sexual reproduction are conserved to the extent that mitotic spore stages have been adapted for meiosis in some taxa (e.g., *Endoraecium*). Further, analysis of the mating type (*MAT*) locus homologues in all suborders of the Pucciniales support a hypothesis of inherent mating compatibility (see Fig 1A and 1B), and the taxonomy of rust fungi is underpinned by morphology of the meiotic stage [5].

Agriculture provided new opportunities for pathogens. Rust fungi with minimal impact in their centres of origin caused epidemics and localised extinctions in new environments on naive hosts, monocultures, and hosts available year-round [6–8]. Sexual reproduction and mutation have driven virulence of rust fungi in agricultural systems [9–11]. A strategy to control heteroecious rust fungi is the removal of alternate hosts to prevent sexual reproduction [10,12]. Nevertheless, genotypic diversity increased in populations where sexual reproduction was restricted [13–16], with rust fungi hypothesised to exchange nuclei between different clones in a process termed somatic hybridisation.

Somatic hybridisation combines the advantages of asexual reproduction by preserving successful haplotypes to minimise recombination load, and of sexual reproduction through new combinations of alleles that may increase fitness, swap deleterious alleles, and evade resistance alleles in hosts. These benefits have implications in managing diseases caused by rust fungi [17].

Somatic hybridisation is an exception to normal life cycles. We discuss several competing hypotheses to explain how shared haplotypes, previously used as evidence to support somatic hybridisation, occur in different genotypes. We provide the minimum criteria needed to support somatic hybridisation over sexual reproduction.

### Evidence for somatic hybridisation in rust fungi

**Fig 2:**
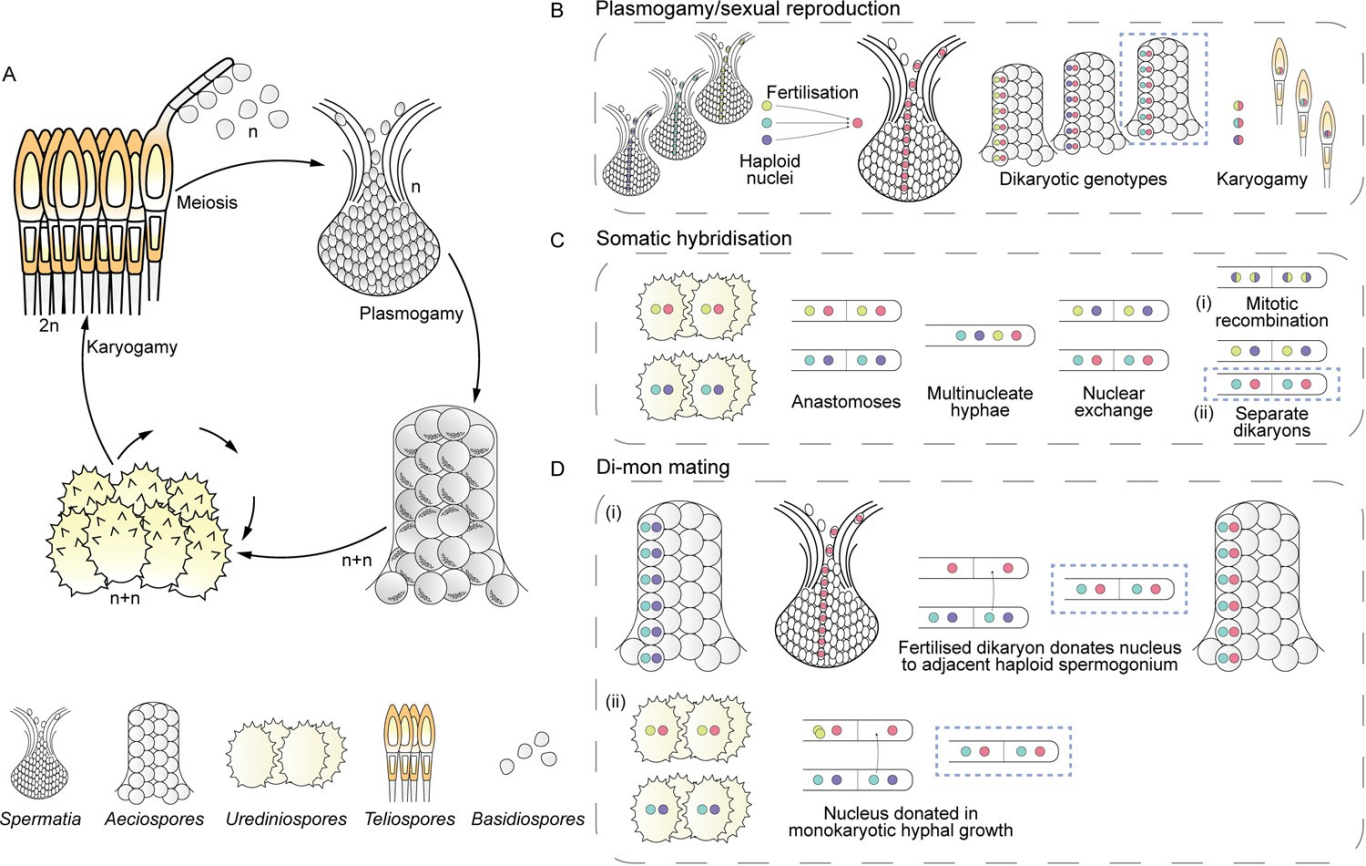
Potential models for exchange of nuclei in life cycles of rust fungi. **(A)** The standard sexual life cycle of macrocyclic, heteroecious rust fungi. **(B)** Exchange of nuclei in sexual reproduction with haplotypes fertilised by spermatia. **(C)** Hypothetical exchange of nuclei between dikaryotic hyphae of rust fungi in somatic hybridisation. **(D)** Hypothetical exchange of nuclei between monokaryotic and dikaryotic hyphae in di-mon mating, whether (i) nuclei from the dikaryotic hyphae of a fertilised spermogonium/aecium are donated to an unfertilised spermogonium; or (ii) nuclei are donated to potential monokaryotic hyphal growth. Multiple pathways in the illustrated figure lead to identical genotypes in blue rectangles.

Rust fungi have complex and plastic life cycles (Fig 2A), with spore stages classified by their ontogeny [18]. Teliospores are the site of karyogamy and meiosis. Basidiospores spread recombinant haplotypes to new hosts. Spermogonia amplify haplotypes by mitosis to fertilise other spermogonia. Aeciospores are produced after plasmogamy and spread new dikaryotic genotypes. Urediniospores are the clonal stage that produce inoculum to spread one genotype. Different spore stages have been lost and gained, or taken on new roles, usually for sexual reproduction, multiple times in the evolution of rust fungi [19,20].

Somatic hybridisation is a process of reassortment without meiosis (specifically, karyogamy, meiosis, and plasmogamy) [21]. A nonexclusive hypothesis is that nuclei in somatic hybrids undergo mitotic recombination in the absence of meiosis [14,17,22]. Mitotic recombination has some of the benefits of sexual reproduction, with changes to genotypic diversity through loss of heterozygosity and structural rearrangements [23].

Park and Wellings [21] reviewed knowledge of somatic hybridisation in rust fungi, highlighting evidence of somatic hybridisation in several taxa. Genome sequencing has since provided evidence of somatic hybridisation in 2 cereal rust fungi. Li and colleagues [24] concluded that identical nuclei in 2 different races of *Puccinia graminis* was evidence of nuclear exchange between 2 dikaryons. Wu and colleagues [25] assembled the nuclei of 2 parents and a putative hybrid of *Puccinia triticina* and hypothesised that the mechanism of somatic exchange was linked to mating loci.

### Somatic hybridisation is best supported with knowledge of phased parental genotypes

Somatic hybridisation is one explanation for the occurrence of identical nuclei in 2 different dikaryotic genotypes of a species [24]. An alternate hypothesis is inherent in the life cycles of rust fungi; one spermogonial haplotype may be fertilised by many spermatia with different haplotypes [26]. In this case, single haplotypes occur in several different genotypes at the same point in time [26] (Fig 2B).

Li and colleagues [24] phased entire nuclei to show they were identical in 2 different genotypes. However, this approach could not reject possibilities that spermogonia donated haplotypes to multiple genotypes through a normal sexual cycle. Ideally, original genotypes of both uredinial parents in a somatic hybrid should be known, and sampled from the point of hybridisation, as designed by Wu and colleagues [25]. The mitotic recombination model can be validated if parental haplotypes are known, as nuclei in hybrids would be mosaics of the 2 nuclei from each parent.

### Opportunities for exchange of nuclei in rust fungi

Rust fungi have limited mycelium and lack clamp connections that ensure fidelity of dikaryotic nuclei in growing hyphae of other Basidiomycota [27]. Somatic exchange may occur from multinucleate hyphae formed by anastomoses [14], as demonstrated in *Phakopsora pachyrhizi* [22]. Di-mon mating, in which monokaryotic tips of hyphae inherit new nuclei from different, dikaryotic mycelia [27], has a similar genetic outcome to somatic hybridisation, except that only one new genotype is produced. Somatic hybridisation and di-mon mating are hypotheses for nuclear exchange among hyphae (Fig 2C and 2D).

### Proposed criteria to determine genotype origin

To distinguish somatic hybridisation or di-mon mating from sexual reproduction, the following guidelines are recommended:

1. reject normal life cycles to show need for somatic hybridisation, such as by absence of an alternate host or haploid life cycle stage;
2. phase haplotypes of putative dikaryotic parents and estimate haplotype frequency in populations;
3. identify phased, reciprocal dikaryotic genotypes as products of nuclear exchange in somatic hybridisation; and
4. reconstruct network genealogy of reassorted and parental haplotypes to show that F1 haplotypes are nested within the genealogies of one haplotype in each parental dikaryon.

Without critical assessment of alternative, and simpler hypotheses in life cycles of rust fungi, we risk misunderstanding pathways to genotypic diversity. Further studies on life cycles are warranted, particularly to explore replication and inheritance of nuclei in haploid stages of rust fungi.
